# Oral cancer and the impact of glossectomy: challenges and pathways to recovery

**DOI:** 10.3389/froh.2026.1761773

**Published:** 2026-04-23

**Authors:** Merna A. Roman, Kamran H. Awan

**Affiliations:** 1 College of Graduate Studies, Roseman University of Health Sciences, South Jordan, UT, United States; 2 College of Dental Medicine, Roseman University of Health Sciences, South Jordan, UT, United States

**Keywords:** cost, glossectomy, mental health, oral cancer, quality of life, speech therapy, treatment

## Abstract

**Objectives:**

Glossectomy is performed to treat and extract the malignant lesions of the tongue. The purpose of the study was to explore the effects of glossectomy on speech and introduction to speech therapy. It also focused on the mental health of patients with oral cancer during and after treatment including issues of financial burden, reentering society, and quality of life post treatment.

**Methods:**

The systematic review was conducted using databases such as PubMed, Scopus, and Web of Science, focusing on studies published between 2001 and 2025. The review included cross-sectional and prospective studies, original research, and review articles. Relevant primary studies were assessed based on predetermined inclusion criteria to ensure alignment with the research objectives.

**Results:**

The reviewed literature showed high-quality studies with minimal bias. Findings indicated that oral cancer patients undergoing partial glossectomy have a better chance for speech retention, facilitated by speech therapy methods. Regarding cost, early-stage intervention with single-modality treatments were less costly than late-stage, multi-modality treatments. Additionally, concerns regarding appearance and reintegration into society were prevalent among participants, with many facing challenges in resuming daily activities post-treatment. Mental health outcomes revealed that more than 40% of patients experienced worsened depression during treatment.

**Conclusion:**

Partial glossectomy is preferred due to its potential for smoother integration to society and improved speech recovery with therapy. Mental health support remains a key focus as oral cancer patients navigate through the ongoing challenges.

**Systematic Review Registration:**

https://mjl.clarivate.com/home

## 1 Introduction

Around 3% of all cancers diagnosed annually account for oral cancer, with an estimated 65,000 people being diagnosed with head and neck cancer in the US alone (1, 2). In fact, oral cancer is the sixth most common malignancy in the world (3). Oral cancer most commonly develops in the mucosa lining the oral cavity and gingiva, the lateral and posterior portions of the tongue, and the back of the oropharyngeal area. Major risk factors include tobacco use, alcohol consumption, human papillomavirus (HPV) infection, excessive sun exposure, genetic predisposition, and advanced age (2). Oral cancer patients come with a unique set of challenging and complex clinical difficulties, the solutions to which can impact both their survival and quality of life (4). Current treatment modalities include surgery, radiation, and chemotherapy, although surgical intervention remains the most common mode of treatment (5). In cases involving the tongue, surgical intervention typically involves excision of both premalignant and malignant lesions, and depending on the severity and stage of cancer, patients may undergo either a partial or full glossectomy.

**Figure 1:**
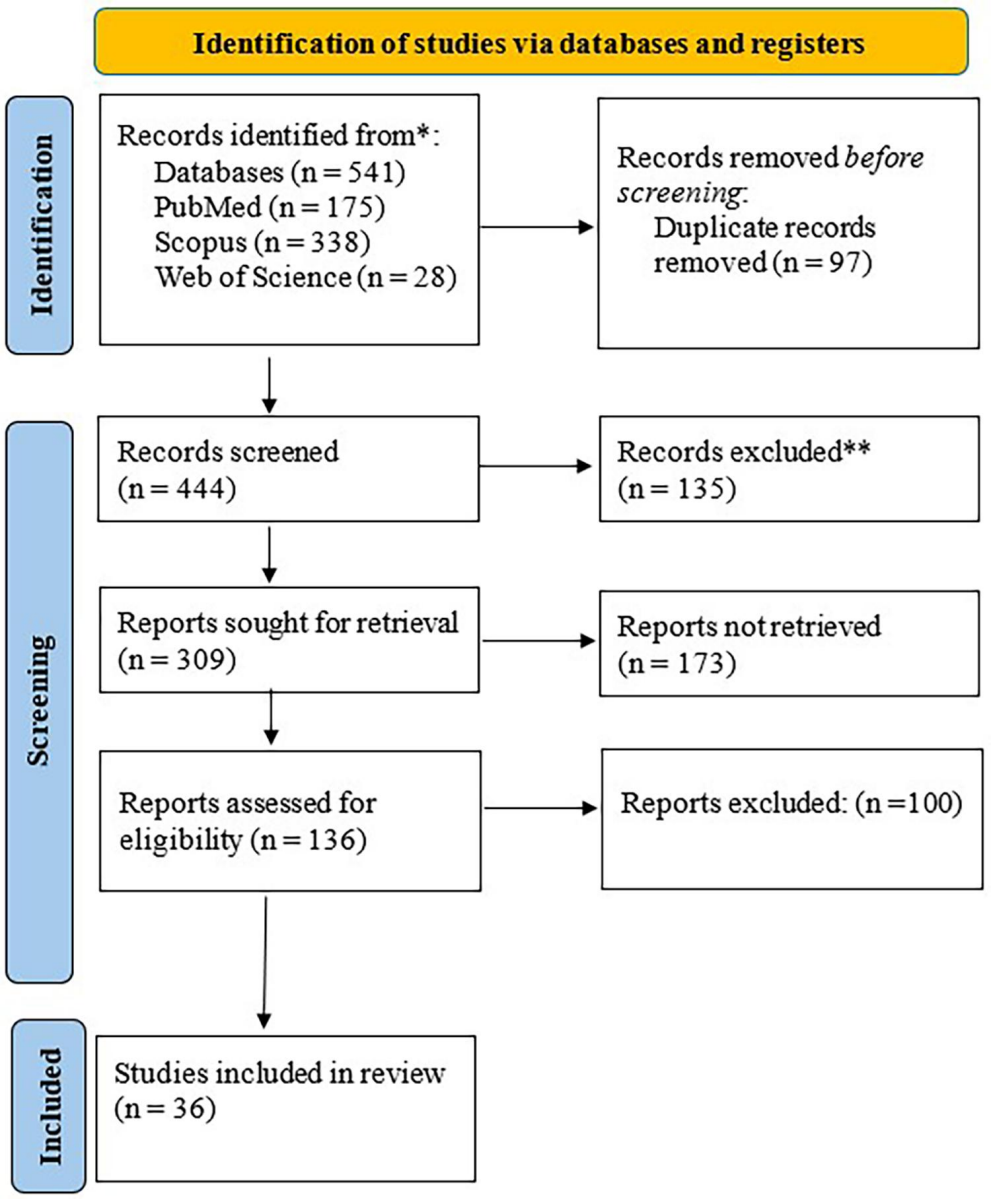
PRISMA diagram.

Patients who undergo partial glossectomy have a better chance of retaining their speech through speech therapy as opposed to patients who undergo full glossectomy. Reentering society poses significant challenges, as patients must adjust to altered speech function and facial esthetics, while also managing financial burdens and mental health challenges. More invasive procedures, such as total glossectomy, are typically associated with greater speech deficits and an increased requirement for rehabilitative interventions. Therefore, early detection of oral cancer is critical as it may require one mode of treatment whereas late-stage cancer often requires multiple treatments resulting in a significant financial burden. An overlooked concern is managing mental health while adapting to new challenges. Depression, anxiety, and stress are common as patients face issues like altered appearance and impaired speech or swallowing, which significantly impact their quality of life. The mouth is vital to an individual’s ability to eat, speak, or interrelate with others; thus, oral cancer treatment is often linked to major physical and psychological burden (6).

Therefore, the purpose of this literature review was to evaluate the effects of glossectomies on oral cancer patients, particularly concerning speech outcomes. It also shed light on the financial burden imposed on patients due to treatment and the effect of treatment on their mental health.

## 2 Methodology

The literature review was conducted using databases such as Web of Science, PubMed, and Scopus, focusing on studies published between 2001 and 2025. The selection process included cross-sectional and prospective studies and original research. Table 1 outlines the keywords used and the number of articles pulled from each database utilized. Keywords that were used to complete this research were “mental health,” “oral cancer,” “cost,” “treatment,” “glossectomy,” “speech therapy,” and “quality of life.” A manual search was also performed to provide background information.

**Table 1:** Search strategy.

Database	Keywords (MeSH Terms)	Results
PubMed	(“Glossectomy”[Mesh] OR “Glossectomy” OR “Glossectomies”) AND (“Cost” OR “Financial Burden” OR “Speech Therapy” OR “Depression” OR “Anxiety” OR “Mental Health” OR “Quality of Life”)	175
	Filters: English AND Publication Date (Last 24 years)	
Scopus	TITLE-ABS-KEY ((“Glossectomy” OR “Glossectomy” OR “Glossectomies”) AND (“Cost” OR “Financial Burden” OR “Speech Therapy” OR “Depression” OR “Anxiety” OR “Mental Health” OR “Quality of Life”)) AND PUBYEAR > 2004 AND PUBYEAR < 2025 AND (LIMIT-TO (LANGUAGE, “English”))	338
	Filters: English AND Publication Date (Last 24 years)	
Web of Science	(“Glossectomy” OR “Glossectomies”) AND (“Cost” OR “Financial Burden” OR “Speech Therapy” OR “Depression” OR “Anxiety” OR “Mental Health” OR “Quality of Life”)	28
	Filters: English AND Publication Date (Last 24 years)	

Relevant primary studies were assessed based on predetermined inclusion and exclusion criteria to ensure alignment with the research objectives. Inclusion criteria included studies that were published in English, available in full text, and within the years 2001–2025. Initially, the process required that the selected articles contain the keywords anywhere in the text. However, this method was later refined to search across title, abstract, and keywords to enhance relevance while being comprehensive and unbiased. Exclusion criteria included any articles published before 2001, were written in a language other than English, and could not be accessed in full text. Table 2 shows the accurate inclusion and exclusion criteria.

**Table 2:** Inclusion & exclusion criteria.

Inclusion Criteria	Exclusion Criteria
From 2001 to 2025 Written in English Full text availability	Any articles published before 2001 Were in a language other than English Could not be accessed in full text

A PRISMA flow diagram was used to assess the eligibility of the articles selected in this review. A database search was performed using MeSH terms and all other necessary limits based on the inclusion and exclusion criteria. Duplicates were removed using a citation management tool such as EndNote. Titles and abstracts of each selected article were then screened and those who did not meet the predetermined criteria were excluded. Subsequently, the articles were reviewed to ensure they were available in full text, and inaccessible articles were removed. Finally, the remaining eligible studies were reviewed in full and included in the literature review.

## 3 Results

The initial search resulted in 541 articles in total. After removing 97 duplicates, 444 records remained. 135articles were subsequently excluded for not including the keywords needed to complete the search. Out of the 309 records, 173 articles were excluded due to inaccessibility to full text. 100 out of 136 records were then excluded based on not meeting the set inclusion and exclusion criteria. 36 articles met the inclusion and exclusion criteria and are included in this literature review.

Eight studies are systematic reviews (6–13), four are original studies (14–17), three are cross-sectional studies (18–20), three are retrospective studies (21–23), five are narrative reviews (1, 2, 5, 24, 25), six are prospective studies (26–31), one is a clinical study (32), two are case-series studies (33, 34), one is a cohort study (35), one is a pilot study (36), one is a review study (37), one is a supplement study (4), one is a longitudinal study (38), and one is a literature review (3). Studies took place in Brazil (14), United Kingdom (10, 18), USA (1, 2, 5, 7, 9, 10, 12, 13, 17, 21, 24, 25, 33, 34, 36, 38), Canada (10, 28, 38), Japan (10), Tokyo (10), India (10, 15, 20, 23, 26, 27, 29, 32, 35, 37), Poland (10), Pakistan (10), Italy (8, 10), Moscow (10), France (10), Iran (10), Malaysia (10), Romania (10), Mexico (10), Delhi (16), Nagoya (30), Seoul (22), Finland (38), Australia (4), Korea (19), Nigeria (13), and China (31). Table 3 outlines the characteristics of the selected articles.

**Table 3:** Characteristics of the selected articles.

Author	Country & Year	Study Design	Primary Outcome	Conclusions
Furia, et al., (14)	**Setting**: Brazil	**Study Type**: Original	To examine the speech intelligibility of patients (before and after speech therapy) who have gone through total, subtotal, or partial glossectomy.	Speech therapy was efficient in progressing speech intelligibility of patients who underwent glossectomy.
	**Period**: 1997-1999	**Sample Size**: 27 patients		
		**Sex**: male (*n* = 24); female (*n* = 3)		
		**Age**: 34–79 years		
Hassanein, et al., (18)	**Setting**: United Kingdom	**Study Type:** Cross- sectional	To explore the psychological outcome of oral cancer patients who have undergone treatment. It also assessed psychological distress such as anxiety, depression and quality of life post-treatment.	Declined functional status and ineffective coping mechanisms are correlated with poor psychological outcomes in oral cancer patients.
		**Sample Size:** 137 patients		
	**Period**: 1992 –1997			
		**Sex:** male (*n* = 47); female (*n* = 21)		
		**Age:** 28–86 years		
Jacobson, et al., (21)	**Setting**: 11 states (United States)	**Study Type:** Retrospective study	To examine the costs of oral cancer across different insurance groups (commercial insurance, Medicare, and Medicaid). It compared the economic burden of treating oral cancers among patients covered by three different insurance types.	The cost of oral cancer is substantial and is the costliest cancer to treat in the United States. This information concerning cost can aid in choosing cost-effectiveness of new technologies and early detention of cancer. The earlier a patient can detect cancer, the less it will cost and there will be a decrease in mortality and morbidity.
	**Period**: 2004–2008	**Sample Size**: 6,812 patients		
		**Sex**: N/A		
		**Age**: 18–85 years		
Moore, et al., (6)	**Setting:** N/A	**Study Type:** Systematic review	To review previous literature discussing quality of life outcomes of oral cancer patients and the different kinds of support needed.	The level of support needed by oral cancer patients is subjective, which shows the nature of the disease and its treatment. There is a need for areas of support relating to oral health and functional impairment, swallowing issues, nutritional issues and psychological concerns that can affect the patient’s overall quality of life.
	**Period:** 2013			
		**Sample Size:** N/A		
		**Sex**: N/A		
		**Age:** N/A		
Omura (5)	**Setting:** United States	**Study Type:** Narrative review	To explain the different treatments of oral cancer. However, surgery remains the mode of treatment.	The most important functions of the oral cavity are mastication, deglutition, maintenance of oral competency, and articulation of speech. When choosing the treatment modality, these factors must be taken into consideration.
	**Period:** 2014	**Sample Size**: N/A		
		**Sex:** N/A		
		**Age**: N/A		
Nanavati, et al., (3)	**Setting:** N/A	**Study Type:** Literature review	To provide an overview of the different etiological agents and risk factors involved in the development of oral cancer.	There is numerous risk factors involved in the development of oral cancer, however the most common are tobacco smoking and betel quid chewing. Despite avoiding this lifestyle, many patients are still diagnosed with oral cancer, which can suggest that genetic susceptibility may contribute to its development.
	**Period:** 2016			
		**Sample Size:** N/A		
		**Sex:** N/A		
		**Age:** N/A		
Saravanan, et al., (20)	**Setting:** India	**Study Type:** Cross-sectional	To assess speech outcome measures such as sounds that are misarticulated and speech intelligibility and its association to the site of the tumor before and after surgery.	Oral cancer patients displayed certain articulation concerns and decreased speech intelligibility when it came to the site of the lesion and the type of reconstruction surgery.
	**Period:** 2016			
		**Sample Size:** 24 patients		
		**Sex:** N/A		
		**Age:** 20–60 years		
Dzioba, et al., (38)	**Setting:** Canada, USA Finland	**Study Type:** Longitudinal study	To explore how oral cancer patients recover their communicative skills, swallowing ability, and quality of life after surgery with or without adjuvant therapy.	Oral cancer patients who undergo surgical resection and reconstruction with or without adjuvant therapy face impairments in both function and quality of life. Clinically, speech was still impaired by the 1 year post assessment.
	**Period:** 2010–2015	**Sample Size:** 117 patients		
		**Sex:** N/A		
		**Age:** 58.2 average years		
Santosh, et al., (10)	**Setting**: USA, Canada, Japan, Tokyo, India, Poland, Pakistan, Italy, Moscow, France, Iran, Malaysia, United Kingdom, Romania, and Mexico	**Study Type**: Systematic review	To place an emphasis on the importance of sign language during the rehabilitation stages in post glossectomy patients as an alternative therapeutic method.	Oral cancer patients experience speech deficits post glossectomy, and rehabilitative therapy involving communication modalities is strongly recommended to better their self-esteem and have a better overall quality of life. Sign language is also a possible alternative therapeutic solution for post glossectomy oral cancer patients.
		**Sample Size**: 3,331 patients		
		**Sex:** N/A		
		**Age:** N/A		
	**Period**: 1980–2015			
Yan, et al., (31)	**Setting:** China	**Study Type:** Prospective study	To evaluate the long-term adjustments in quality of life (QOL) in oral cancer patients and to study the potential factors that predicted quality of life at 8 years posttreatment.	On average, quality of life was generally favorable in long term for oral cancer patients, especially with early detection and were treated only with primary surgery. It should be noted that some persistent treatment concerning oral dysfunction (speech, chewing, taste), shoulder disability, and appearance was necessary to improve long-term quality of life.
	**Period:** 01–08/2005			
		**Sample Size:** 71 patients		
		**Sex:** Male (*n* = 38); female (*n* = 33)		
		**Age:** 35–90 years		
Jain, et al., (16)	**Setting**: Delhi	**Study Type**: Original	To examine the speech and swallowing impairment after partial glossectomy in oral cancer patients. To put an emphasis on the role of speech and swallowing rehabilitation exercises post-treatment.	Post-treatment speech and swallowing exercises can be a crucial step for improving the patient’s overall quality of life. These exercises are simple and can be practiced during post-treatment follow-ups and at home.
	**Period**: 2016–2018			
		**Sample Size**: 50 patients		
		**Sex**: male (*n* = 32); female (*n* = 18)		
		**Age**: 30–68 years		
Kumar, et al., (27)	**Setting**: North India	**Study Type**: Prospective	To evaluate the levels of depression, anxiety, and stress at three different time points which were at diagnosis, one month after treatment, and three months after treatment.	Depression and stress increased after treatment while anxiety became stable. The results of this study raise awareness among surgeons to take psychological needs of oral cancer patients into account.
	**Period**: 2013–2016	**Sample Size**: 111 patients		
		**Sex**: male (*n* = 62); female (*n* = 13)		
		**Age**: 18–60 + years		
Valdez, et al., (25)	**Setting:** North Carolina	**Study Type:** Narrative review	To explore how treatment of oral cancer can have adverse effects on esthetics, speech, voice, and swallowing. These effects can lead to negative impacts on the quality of life of oral cancer patients.	Numerous aspects of a patient’s life, including psychosocial, physical, and financial are affected by various stages of oral cancer. This can play a major role in contributing to the negative or positive effects in the patient’s overall quality of life.
		**Sample Size:** N/A		
	**Period:** N/A	**Sex:** N/A		
		**Age:** N/A		
Wong, et al., (4)	**Setting:** Australia	**Study Type:** Supplement	To highlight the epidemiology and risk factors for oral cancer in Australia, the different clinical presentations and the classification stages.	Oral cancer is challenging with high mortality rates; therefore, prevention through education regarding smoking and alcohol consumption is crucial. Early detection, ongoing surveillance, and follow up are some of the different role dentists can manage for oral cancer patients.
	**Period:** 2018	**Sample Size:** N/A		
		**Sex:** N/A		
		**Age:** N/A		
Elting, et al., (7)	**Setting:** Texas	**Study Type:** Systematic review	To explore how oral complications of cancer treatment are common and costly but they are rarely discussed.	Oral complications of oral cancer treatment are common and can cause morbidity which include xerostomia, oral infections, acute and chronic oral pain, osteoradionecrosis and many more serious challenges. All of these costs accumulate and create a financial burden on oral cancer.
	**Period:** N/A			
		**Sample Size:** N/A		
		**Sex:** N/A		
		**Age:** N/A		
Inchingolo, et al., (8)	**Setting:** Italy	**Study Type:** Systematic review	To provide an understanding of the evolution of oral cancer over the years.	Oral surgeons and oncologists must learn more about the history of treating oral cancer to avoid past unsuccessful efforts.
	**Period:** N/A			
		**Sample Size:** N/A		
		**Sex:** N/A		
		**Age:** N/A		
Pyne, et al., (28)	**Setting**: Canada	**Study Type**: Prospective	To examine self-reported functional and quality of life data in patients who underwent total glossectomy with preservation of the larynx (TGLP) and free flap reconstruction.	Significant reduction in functional measurements were found in patients undergoing total glossectomy. However, patients reported stable functional and quality of life post-treatment.
	**Period**: 2009–2017	**Sample Size**: 22 candidates		
		**Sex**: male (*n* = 11); female (*n* = 5)		
		**Age**: 28–73 years		
Rogers, et al., (17)	**Setting:** United States	**Study Type:** Original	To provide data on a 10-year health related quality of life outcomes after oral cancer treatment.	Post oral cancer treatment, most patients reported good to better overall quality of life long-term. Changes between 2 and 10 years were small, however, there were some clinically significant improvements in appearance, chewing, mood, and anxiety. Changes at an individual level were not so constant.
	**Period**: 1992–2004	**Sample Size:** 674 patients		
		**Sex:** male (*n* = 426); female (*n* = 248)		
		**Age:** 55–75 + years		
U.S. DEPARTMENT OF HEALTH AND HUMAN SERVICES National Institutes of Health (2)	Setting: United States	**Study Type:** Narrative review	To explore the causes and provide an overview of oral cancer. To also provide more information about the treatment options of oral cancer.	Some of the risk factors of oral cancer are HPV, age, genetics, tobacco and alcohol use. The most common treatment option for oral cancer is surgery and possibly radiation therapy and chemotherapy. Oral cancer in its later stages may need a combination of treatments.
	Period: 2020	**Sample Size:** N/A		
		**Sex:** N/A		
		**Age:** N/A		
Bhattacharya, et al., (32)	**Setting**: India	**Study Type**: Clinical study	To analyze the speech outcomes of oral cancer patients who underwent treatment with reconstruction of the tongue.	Patients with oral tongue cancer can accomplish good-to-acceptable speech outcomes and post therapy. Correlation between speech intelligibility scores and the type of glossectomy were found.
	**Period**: 2013–2014			
		**Sample Size**: 69 patients		
		**Sex**: male (*n* = 45); female (*n* = 24)		
		**Age**: 29–81 years		
Das, et al., (37)	**Setting:** India	**Study Type:** Review	To demonstrate the dramatic increase in oral cancer all around the world but especially in India which has the greatest number of oral cancer cases.	India has an alarming number of oral cancer cases and continues to increase. There is an urgent need to raise awareness concerning the growing burden of oral cancer and immediate intervention is required to decrease the incidence rate and reduce mortality.
	**Period:** 2021			
		**Sample Size:** N/A		
		**Sex:** N/A		
		**Age:** N/A		
Singh, et al., (29)	**Setting**: India	**Study Type:** Prospective	To determine the healthcare costs of oral cancer in India and to create repeatable costing model that can be used for other cancers and in other countries.	Oral cancer remains a global burden in India which is worsened by the inability to afford treatment. The average cost of treatment increased by 44.6% with multiple modalities of treatment compared to surgery alone.
	**Period**: 2019–2020			
		**Sample Size:** 100 patients		
		**Sex:** male (*n* = 87); female (*n* = 13)		
		**Age**: 24–65 years		
Dasgupta, et al., (26)	**Setting:** India	**Study Type**: Prospective study	To examine speech in oral cancer patients following hemiglossectomy with primary closure and radiotherapy.	Articulatory errors increased post hemiglossectomy and radiotherapy. However, the number of errors decreased over time. With adequate speech therapy, oral cancer patients can regain their preoperative articulation.
	**Period:** 2019–2021			
		**Sample Size**: 20 patients		
		**Sex**: male (*n* = 14); female (*n* = 6)		
		**Age:** 50–60 years		
Goswami, et al., (15)	**Setting:** India	**Study Type:** Original	To analyze the financial burden on oral cancer patients.	Oral cancer patients who are diagnosed in advanced stages tend to have more than one treatment which can be more costly. On the other hand, early detection alleviates the treatment costs, however, it should go hand in hand with early detection.
	**Period:** 2015–2017			
		**Sample Size:** 100 patients		
		**Sex:** male (*n* = 81); female (*n* = 19)		
		**Age:** 30–72 years		
Hagedorn, et al., (36)	**Setting:** United States	**Study Type:** Pilot study	To investigate whether speakers who have undergone partial glossectomy employ different compensatory strategies depending on the targeted manner of articulation.	Compensatory approaches utilized by oral cancer patients who have undergone glossectomy to produce target alveolar segments can differ as a function of target manner of articulation in a subtle yet significant ways.
	**Period:** 2022	**Sample Size:** 2 patients		
		**Sex:** male (*n* = 1); female (*n* = 2)		
		**Age:** 52; 70		
Ribeiro-Rotto, et al., (11)	**Setting:** N/A	**Study Type:** Systematic review	To provide a thorough systematic review on the economic burden of oral cancer worldwide.	The economic burden of oral cancer is both substantial and underestimated. More cost of illness studies are still needed especially with direct and non-direct medical costs.
	**Period:** 2001–2021			
		**Sample Size:** N/A		
		**Sex:** N/A		
		**Age:** N/A		
Burnham, et al., (34)	**Setting**: Georgia	**Study Type**: Case series	To provide data on total glossectomy with total laryngectomy patients’ survival and functional outcomes.	Total glossectomy is associated with high mortality. Speech intelligibility was eliminated and patients had to rely on alternative methods of communication. Quality of life should be discussed prior to performing this procedure.
	**Period**: 2014–2021			
		**Sample Size**: 39 patients		
		**Sex**: male (*n* = 28); female (*n* = 11)		
		**Age:** 62.7 years		
Amechi, et al., (33)	**Setting**: Georgia	**Study Type**: Case series	To discuss the quality of life of oral cancer patients post total glossectomy.	There is minimal literature on the quality of life of oral cancer patients due to high mortality rates. It is critical for patients to be educated on the psychological and social implications when proceeding with a total glossectomy.
	**Period**: 2014–2021			
		**Sample Size**: 39 patients		
		**Sex**: N/A		
		**Age**: N/A		
Suzuki, et al., (30)	**Setting:** Nagoya	**Study Type**: Prospective study	To examine the changes in the quality of life and psychological distress of oral cancer patients undergoing total and hemi glossectomies.	12 months after surgery, oral cancer patients who underwent total/subtotal glossectomy had worse scores for quality of life and depression than those who underwent a hemiglossectomy.
	**Period:** 2007–2019	**Sample Size**: 43 patients		
		**Sex**: male (*n* = 30); female (*n* = 12)		
		**Age**: 43.6–75.3 years		
Russo, et al., (12)	**Setting:** United States	**Study Type**: Systematic review	To evaluate the functional outcomes and obstacles of total glossectomy with laryngeal preservation and reconstruction with free or pedicled flaps.	Total glossectomy with laryngeal preservation and pedicled or free flaps reconstruction may result in good functional outcome and an acceptable qualify of life.
	**Period:** 1985–2020	**Sample Size**: 642 patients		
		**Sex**: male (*n* = 488); female (*n* = 154)		
		**Age**: 54.2 years		
Chen (1)	**Setting**: United States	**Study Type:** Narrative review	To provide an insight into how common oral cancer is and the survival rate depending on the stage. To describe how cancer attacks one’s cells and overall immune system. To present some of the risk factors of oral cancer such as HPV, smoking, and genetics.	It was found that the more a person smoked or vaped, the more DNA damage that was found which can cause changes potentially relevant to oral cancer. Oftentimes, oral cancers can go undetected until it is advanced and difficult to treat which decreases the survival rate.
	**Period**: 2024	**Sample Size:** N/A		
		**Sex**: N/A		
		**Age**: N/A		
Jimenez- Labaig, et al., (9)	**Setting:** United States	**Study Type**: Systematic review	To examine the mental health of oral cancer patients.	Head and neck cancer patients presented a notable prevalence of mental health concerns. Suicide remains a concern among patients.
	**Period:** 2023	**Sample Size**: N/A		
		**Sex**: N/A		
		**Age**: 60.7 years		
Kim, et al., (22)	**Setting:** Seoul	**Study Type:** Retrospective study	To examine post hemiglossectomy speech outcomes by considering the reconstruction type and other factors.	Acceptable speech outcomes were observed in post hemiglossectomy with free flap reconstruction.
	**Period:** 2017–2022			
		**Sample Size:** 24 patients		
		**Sex:** male (*n* = 14); female (*n* = 10)		
		**Age:** 53.3 years		
Lee, et al., (19)	**Setting:** Korea	**Study Type:** Cross-sectional	To explore the association of mental and oral health with the health-related quality of life in both patients with cancer and survivors.	Patients with cancer scored lower in health-related quality of life than cancer survivors and showed significant differences. There is a need to implement policies for each group to improve overall quality of life.
	**Period:** 2005–2018	**Sample Size:** 3271 patients		
		**Sex**: male (*n* = 1238); female (*n* = 2033)		
		**Age:** ≥19 years		
Bigcas, et al., (24)	**Setting**: Nevada	**Study Type**: Narrative review	To explain glossectomy and the differences between partial, hemi, subtotal, and total glossectomies. To also compare the different techniques for performing a glossectomy.	Surgery is the recommended and most common method of treatment for oral tongue cancer. Comprehending the disadvantages and advantages of the many approaches to glossectomy can aid in the appropriate counseling to patients.
	**Period**: N/A			
		**Sample Size**: N/A		
		**Sex**: N/A		
		**Age**: N/A		
Dwivedi, et al., (35)	**Setting:** India	**Study Type:** Cohort study	To evaluate the rehabilitation results of Indian patients who have undergone partial or hemiglossectomy and focusing on speech therapy.	This study emphasized the importance of a multidisciplinary approach to rehabilitation, highlighting the roles of motivation, family support, and early-stage diagnosis in successful recovery.
	**Period:** 2021–2022			
		**Sample Size:** 55 patients		
		**Sex:** male (*n* = 37); female (*n* = 18)		
		**Age:** 52.3 average years		
Nemade, et al., (23)	**Setting:** India	**Study Type:** Retrospective	To explore quality of life evaluation of long term (more than 5 years) for total glossectomy survivors.	Total glossectomy patients reported favorable five-year long-term outcome in quality of life despite moderate to severe impairments in speech and swallowing. Also, they displayed critical challenges in speech concerning intelligibility, articulation, and overall speech impairment.
	**Period:** 2014–2017			
		**Sample Size:** 25 patients		
		**Sex:** N/A		
		**Age:** N/A		
Tundealao, et al., (13)	**Setting:** United States and Nigeria	**Study Type:** Systematic review	To evaluate pre- and post-operative changes in the quality of life of tongue cancer patients who underwent glossectomy.	Following glossectomy, oral cancer patients can preserve certain aspects of quality of life especially in functional domains such as swallowing, speech, and taste. However, quality of life outcomes differs depending on the extent of glossectomy and the type of reconstruction.
		**Sample Size:** 2,832 patients		
	**Period:** 04/2025			
		**Sex:** male (*n* = 1,756); female (*n* = 1,076)		
		**Age:** 55.1 average years		

## 4 Discussion

### 4.1 Speech After Glossectomy

Speech is one of the most powerful and universal social interaction tools used in society.^2^ It plays a crucial role in one’s day-to-day activities. The tongue remains one of the most essential articulators during the production of vowels and many consonant sounds (14). This is why an effect on the tongue, whether pathological or surgical, can have a profound impact on the individual and their speech. The degree, extent, and location of the resection influence the resulting speech impairment (14). In cases where part of the tongue’s volume and mobility can be preserved, the chances of successful rehabilitation are usually higher (14).

Cancer of the tongue can bring about concerns regarding speech in patients. For instance, if the lesion is found in the anterior portion of the tongue, or in the posterior aspect of the tongue, then articulation would be disturbed (20). Crucial aspects that determine the effect on speech consist of quantity of tissue extracted during surgery, frequency of speech/swallowing treatment and patient compliance. In a cross-sectional study, speech outcome measures such as sounds that are misarticulated and speech intelligibility, and its association to the tumor site pre- and post-surgery was assessed. Both voice quality and resonance were compromised post-surgery due to the alterations in the oral cavity volume. Articulation was also altered due to the tongue being unable to assume the normal position to provide valuing action that is necessary for precise articulation (20). Results showcased that speech outcome relied on the site of lesion as oral cancer patients tend to have more articulation mistakes and fewer speech intelligibility.

In a cross-sectional study, speech outcomes after glossectomy using an intelligibility assessment tool and adjuvant therapy were assessed. This assessment tool contained five components: vowel intelligibility (VI), consonant intelligibility (CI), word intelligibility (WI), paragraph intelligibility (PI), and an overall speech intelligibility score (OSIS). Speech intelligibility was defined as the percentage of the component understandable to the listener. The evaluation speech outcomes were performed at an average of 14 months post treatment of oral cancer. Results showed that VI, CI, WI, PI were approximately 99.27%, 86.86%, 85.52%, and 88.72%, respectively (32). Examining OSIS showed that 78.3% of patients had normal speech, while 21.7% had an acceptable outcome. No patients showed any signs of incomprehensible speech. The primary determinants of a good speech outcome after glossectomy are still not well established.

### 4.2 Psychological Distress and Quality of Life

Mental health concerns among oral cancer patients are often overlooked. However, when patients are first diagnosed, they experience various physical, mental, emotional, and social difficulties. The psychological burden oftentimes occurs due to the rapid changes in daily activities such as chewing, swallowing, and speech. Dissatisfaction with one’s postoperative appearance and other aesthetic factors can lead to a poor psychological outcome.

While many studies focus on depression and anxiety, they do not account for stress as often. A study by Kumar, et. al (27), analyzed data from 2013 to 2016 in India, and it evaluated three main domains: depression, anxiety, and stress. These domains were assessed at three different time points: at the time of diagnosis, one month after treatment, and three months after discharge. Depression levels increased from time of diagnosis to three months after discharge, while anxiety remained stable across all time points. In contrast, stress seemed to have a stable start from time of diagnosis but increased between the one-month and three-month after discharge periods. According to the study, depression was reported in more than 40% of patients during treatment (27).

Another study done by Hassanein, et al. (18),, examined the high prevalence of depression and anxiety among oral cancer patients and the effectiveness of coping mechanisms. The study reported a strong association between ineffective coping strategies such as helplessness/hopelessness, and anxious preoccupation and depression and anxiety. It was found that 16% of patients experienced anxiety, 19% of patients experienced depression, and 25% of oral cancer patients experienced both anxiety and depression (18). It is important to note that oral cancer patients often come from a lower socio-economic backgrounds and are usually less educated, therefore, they are more susceptible to psychological distress compared to patients with other types of cancer (18).

A study by Yan, et al. (31), examined long-term changes in quality of life (QOL) in oral cancer patients and the potential factors that predicted quality of life at 8 years posttreatment. It should be noted that quality of life was found to be better among oral cancer survivors compared with non-survivors at the 1-year follow-up. Levels of pain, mood, and anxiety displayed noteworthy improvements, both clinically and statistically, between diagnosis and at the 8-year mark posttreatment. However, chewing, speech, and oral dysfunction (chewing, speech, and taste) deteriorated during this specific studied period. This is explained by the fact that treatment relieved pain, and long-term survivorship or enormous level of social support from families aided patients coping with depression resulted by the disease. However, the oral dysfunction and difficulties with appearance were often the result of treatment (31). In general, quality of life was found to be promising in patients who detected oral cancer in its early stages and were only treated with primary surgery.

### 4.3 Financial Burden

A study by Singh, et al. (29), analyzed data from October 2019 and March 2020 in India and determined that the cost of treatment at an advanced stage was 42% greater than early stages. The average cost of treatment increased by 44.6% with multiple modalities of treatment compared to surgery alone. It is important to note that there have been substantial advances in the diagnosis and management of oral cancer in the recent years, but these advances have added to the increased costs of treatment which can significantly impact the cost of care for both providers and patients. Most oral cancer patients that receive any form of treatment are usually left unemployed due to the incapacitating process of the disease, which results in them becoming an economic and psychological burden on their friends and family. Patients with health insurance or government aid are also not immune to the cost of care because they are usually unaware of the actual cost of treatment. This indicates high out of pocket expenses which imposes significant amounts of debt on patients and their families. Oral cancer patient’s costs accumulate from over-the-counter medications, co-payments, deductibles, time costs, transportation costs, productivity costs, and lost wages (7).

Up to 18% of tongue carcinomas are diagnosed at an advanced stage, requiring total glossectomy (12). A study by Jacobson, et al. (21), found that individuals with commercial insurance faced average medical costs of $79,151 in the first year after an oral cancer diagnosis. This is significantly higher than the costs of treating other cancers, which range from $31,559 to $65,123. Costs for individuals undergoing surgery, radiation, and chemotherapy averaged to be $153,892 during the year after diagnosis. These higher costs are expected due to the various treatment modalities required for late or advanced-stage diagnoses. It is important to note that patients with more severe oral cancer may have had to stop working, which would add to the indirect costs of treatments and follow-up appointments. The side effects of treatment, such as fatigue and nausea, can limit the number of hours a patient is able to work. It is preferred that oral cancer is detected at an earlier stage, as it could result in a single modality treatment, rather than multiple modalities of treatment (21).

## 5 Conclusion

It is preferred that oral cancer is detected at an earlier stage, as it could result in less invasive surgery and in a single modality treatment, rather than multiple modalities of treatment. More invasive procedures, such as total glossectomy, are associated with greater speech deficits and an increased requirement for rehabilitation interventions. Therefore, partial glossectomy is preferred due to its potential for smoother integration into society and improved speech recovery with therapy. Depression, anxiety, and overall mental health support remain a key focus as oral cancer patients navigate through the ongoing challenges.

## Data Availability

The original contributions presented in the study are included in the article/Supplementary Material, further inquiries can be directed to the corresponding author.
